# Integrative analysis of transcriptomic data related to the liver of laying hens: from physiological basics to newly identified functions

**DOI:** 10.1186/s12864-019-6185-0

**Published:** 2019-11-07

**Authors:** Audrey Gloux, Michel J. Duclos, Aurélien Brionne, Marie Bourin, Yves Nys, Sophie Réhault-Godbert

**Affiliations:** 1 BOA, INRA, Université de Tours, 37380 Nouzilly, France; 2 Institut Technique de l’Aviculture (ITAVI), Centre INRA Val de Loire, F-37380 Nouzilly, France

**Keywords:** Oligoarray data, Hen, Sexual maturity, Liver, Physiology, Metabolism, Reproduction

## Abstract

**Background:**

At sexual maturity, the liver of laying hens undergoes many metabolic changes to support vitellogenesis. In published transcriptomic approaches, hundreds of genes were reported to be overexpressed in laying hens and functional gene annotation using gene ontology tools have essentially revealed an enrichment in lipid and protein metabolisms. We reanalyzed some data from a previously published article comparing 38-week old versus 10-week old hens to give a more integrative view of the functions stimulated in the liver at sexual maturity and to move beyond current physiological knowledge. Functions were defined based on information available in Uniprot database and published literature.

**Results:**

Of the 516 genes previously shown to be overexpressed in the liver of laying hens, 475 were intracellular (1.23–50.72 fold changes), while only 36 were predicted to be secreted (1.35–66.93 fold changes) and 5 had no related information on their cellular location. Besides lipogenesis and protein metabolism, we demonstrated that the liver of laying hens overexpresses several clock genes (which supports the circadian control of liver metabolic functions) and was likely to be involved in a liver/brain/liver circuit (neurotransmitter transport), in thyroid and steroid hormones metabolisms. Many genes were associated with anatomical structure development, organ homeostasis but also regulation of blood pressure. As expected, several secreted proteins are incorporated in yolky follicles but we also evidenced that some proteins are likely participating in fertilization (ZP1, MFGE8, LINC00954, OVOCH1) and in thyroid hormone maturation (CPQ). We also proposed that secreted proteins (PHOSPHO1, FGF23, BMP7 but also vitamin-binding proteins) may contribute to the development of peripheral organs including the formation of medullar bones to provide labile calcium for eggshell formation. Thirteen genes are uniquely found in chicken/bird but not in human species, which strengthens that some of these genes may be specifically related to avian reproduction.

**Conclusions:**

This study gives additional hypotheses on some molecular actors and mechanisms that are involved in basic physiological function of the liver at sexual maturity of hen. It also revealed some additional functions that accompany reproductive capacities of laying hens, and that are usually underestimated when using classical gene ontology approaches.

## Background

Similarly to other animal species, reproduction of bird females is regulated by the hypothalamus-pituitary-gonads axis, that secretes a cascade of hormones stimulated by internal factors of the juvenile females (physiology, life cycle, overall health and access to food) but also by external factors including environmental temperature and photoperiod (which determines the onset of egg production and synchronizes the daily reproductive cycle) [1]. Sexual maturity of hens begins with the production of gonadotropin-releasing hormone (GnRH) by the hypothalamus, which consequently stimulates the production of luteinizing hormone (LH) and follicle-stimulating hormone (FSH) by the pituitary gland. These interrelated hormones will trigger the synthesis of gonadal steroids (estradiol, testosterone, progesterone) by thecal and granulosa cells that support the growth of yolky follicles in the ovary [2–4]. All these hormones are regulating the development and the ovulation of the preovulatory follicle (F1 follicle), whose maturation therefore relies on feedback signals between gonads and the hypothalamus-pituitary axis. In birds (in contrast to mammals), this neuroendocrine system controlling egg production and supporting embryonic development of offspring stimulates the expression of hormone-dependent genes, not only in the reproductive oviduct and ovary but also in other vital organs/tissues such as the liver, which synthesizes the majority of yolk components [5]. This hepatic gene expression supports many lipid changes associated with the development of reproductive organs, including egg yolk formation and supporting tissues. In addition, sexual maturity affects a variety of other traits in the chicken including secondary sexual characteristics such as the comb size that is a sexual ornament [6]. Among other changes, laying hens undergo major modifications in their bone structure. The high concentration of estrogen in combination with testosterone changes the function of osteoblasts to produce the medullary bone that provides a labile source of calcium for eggshell formation [7]. In this respect, it has been demonstrated that osteogenic cells on the surface of medullary bone express estrogen alpha receptors [8–10].

Using a 20 K chicken oligoarray, a total of 582 probes were shown to be over-expressed in the liver of 38-week sexually mature hens versus 10-week juvenile hens (Layer ISA brown, Hendrix Genetics, 1.2 to 67 fold-differences) [11]. The integrative analysis of these results were not published, because the authors chose to focus on proteases and antiproteases that were overexpressed in relation to the activation of egg yolk precursors, egg yolk formation and fertilization. More recently, RNA-Seq analysis on total RNA harvested from the liver of 20 week-old juvenile hens and 30 week-old laying hens (Lushi green shell chickens) revealed 1082 up-regulated genes in sexually mature hens [12]. The gene ontology term analysis of these data showed that the differentially expressed genes were significantly enriched in oxidation reduction, sterol and cholesterol metabolic processes, and lipid biosynthetic processes. From these results, the authors concentrated their discussion on the metabolic pathways associated with lipid metabolism [12]. These two publications highlight the difficulty of exhaustively addressing the physiological functions associated with data obtained from high throughput methods. Thus, the objective of the present article was to give an integrative and straightforward overview of the functions related to the proteins that were shown to be overexpressed in the liver at sexual maturity of hens. For this purpose, we selected and used the data obtained from a layer line that is used worldwide [11]. The originality and the added-value of the present work is that the functional annotation of overexpressed genes was achieved using a manual approach by retrieving information from Uniprot database that is currently available, but also by considering known physiological changes associated with sexual maturity in hens. The reason for such an approach instead of using classical gene ontology analysis is that most gene ontology tools are highly efficient to decipher the biological functions of proteins and molecules responsible for physiopathological situations in mammals. In contrast, these approaches are clearly less relevant when using oviparous models. This comment is particularly true for the chicken liver of females knowing that this organ expresses and secretes in blood most precursors of yolk proteins that lack homologous genes in mammals. Consequently, the functional annotation of such proteins is very limited, although these molecules are of major physiological importance. Moreover, sexual maturity in hens induces many physiological and metabolic changes that cannot be transposed to mammals (comb development, bone remodeling, egg formation). To give this integrative view of the functions associated with the liver at sexual maturity of hens, we distinguished proteins that are confined to the liver (membrane/cell localization) from those that are secreted in the blood stream such as yolk precursors, and others that may have a more systemic effect and/or an effect at another physiological site than the liver.

## Results

### Features of overexpressed genes

#### Number of overexpressed genes

Using a 20 K chicken oligoarray corresponding to 12,595 different chicken probes, a total of 582 probes had been shown to be over-expressed in the liver at sexual maturity of hens [11] (Additional file 1, column A-C). It is noteworthy that these genes have a basal expression in the liver of immature pullets. Further to this publication, the Gallus_gallus-4.0 assembly was released in April 2013 by the International Chicken Genome Consortium followed by the Gallus_gallus-5.0 assembly (released in Oct 2016) and GRCg6a (GCA_000002315.5) assembly, submitted by the Genome Reference Consortium on April 2018. Using this last genome annotation, we re-analyzed the full list of genes shown to be overexpressed in the liver of mature hens to remove redundancies and to update accession numbers. This preliminary work allowed us to restrict the initial list of 582 genes to 516 genes (Additional file 1) that are overexpressed in the liver of laying hens (1.2 to 67-fold changes). Twelve were withdrawn (Additional file 1, column I, lines 4–15) from databases and a total of 54 genes were found to be redundant (Additional file 1, lines 532–585).

#### Distribution between secreted/non secreted proteins

**Fig. 1:**
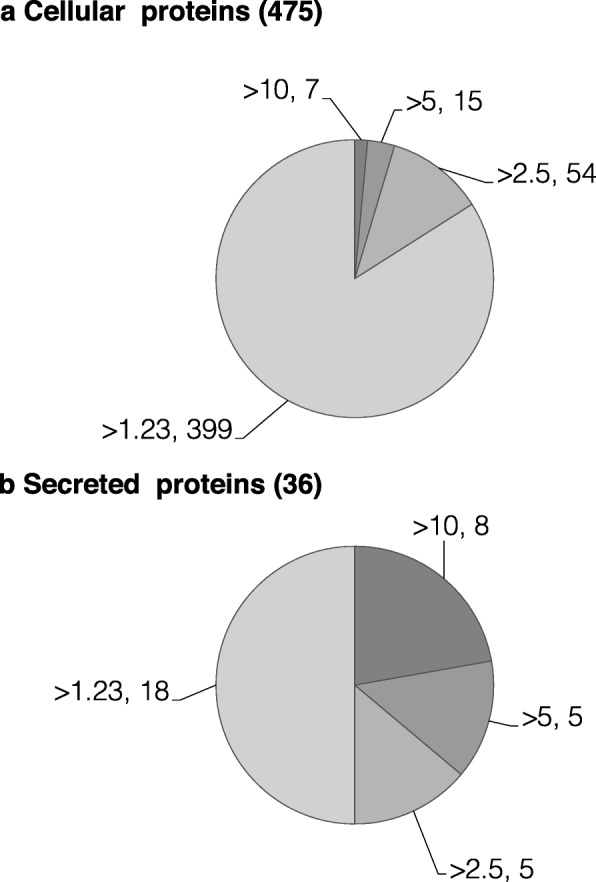
Differential of expression of intracellular (**a**) and secreted (**b**) proteins. For each category, the first number corresponds to the level of overexpression and the second to the number of associated genes

Some expressed proteins are secreted in the blood stream to support physiological processes at distant sites (including vitellogenesis at the ovary site) while others are restricted to the intracellular compartment of the liver. To better appreciate the intrinsic role of each candidate, we retrieved information related to secreted and non-secreted proteins (information available from Uniprot website) assuming that their respective role may be different and/or complementary. Of the 516 overexpressed genes, 475 were intracellular or localized in the plasma membrane (1.23–50.72 fold changes when comparing with immature livers of pullets), while only 36 were predicted to be secreted, as they possess a signal peptide (1.35–66.93 fold changes), and 5 had no related information on their cellular location (1.41–3.03 fold changes) (Fig. 1, Additional file 2, columns E, F). Altogether, these data reveal that most overexpressed genes (a total of 475 genes) are confined to the liver organ (either intracellular or anchored in cell membranes). Thereby, a total of only 36 proteins may be directly secreted in the blood stream as precursors of yolk components (vitellogenins, vitamin-binding proteins etc.) that are incorporated in the growing follicles in the ovary or to play a regulatory role at other distant physiological sites.

#### Number of bird-specific genes

Thirteen overexpressed genes with fold changes ranging from 1.66 to 66.92 had no identified homologs in mammals (Table 1, Additional file 2, column G), suggesting a specific role in relation to sexual maturity of chicken females and/or reproduction. These bird-specific genes are distributed all along the chicken genome and localized within 8 different chromosomes. They include avidin-related protein 6-like (fold change =10.5) that is localized in the W chromosome (female-specific chromosome).

**Table 1:** Chicken genes lacking mammalian homologs. ML/IL ratio: ratio of gene expression in the liver of 38-week laying hens (ML) to the expression in the liver of 10-week juvenile pullets (IL)

Name [*Gallus gallus*]	Gene symbol/Gene ID/chromosomic localization	ML/IL Ratio	Subcellular location/ short resume of biological functions
riboflavin-binding protein	RBP/396449/Chr8	66.92	Secreted/Vitamin metabolism
vitellogenin-3	VTG3/424534/Chr8	45.88	Secreted/Ion metabolism
apovitellenin-1	APOV1/396476/Chr1	42.38	Secreted/Lipase inhibitor
cathepsin E-A-like	CTSEAL/417848/Chr1	34.85	Secreted/Proteolysis
vitellogenin-1	VTG1/424547/Chr8	33.79	Secreted/Ion metabolism
family with sequence similarity 20, member C-like	FAM20CL/418020/Chr1	11.44	Cell/Ion metabolism (calcium)-Biomineralization
avidin-related protein 6-like	LOC426220/426220/ChrW	10.48	Secreted/Vitamin and cofactor metabolisms
microsomal triglyceride transfer protein-like	MTTPL/769580/Chr6	7.65	Secreted/Lipid metabolism
sulfotransferase	SULT/395933/Chr3	5.47	Cell/?
probable 2-ketogluconate reductase-like	2KTGRL/100858664/Chr2	1.71	Cell/?
fibronectin type III domain containing 3A-like	FNDC3AL/422151/Chr4	1.59	Membrane/?
A-kinase anchor protein 17B-like	LOC422372/422372/Chr4	1.37	Cell/Protein metabolism
serine/arginine-rich splicing factor 5a	SRSF5A/423265/Chr5	1.36	Cell/?

### Functions associated with overexpressed genes

Sexual maturation in birds implies profound physiological modifications, contributing to and accompanying the onset of reproductive functions. The liver is a very important actor of sexual maturity and consequently, it undergoes many physiological and metabolic changes in response to hormone stimulation. However, as mentioned above, it seems important to distinguish proteins confined to the liver (cellular proteins) from those that are secreted and that are susceptible to play a role in another organ/tissue.

**Fig. 2:**
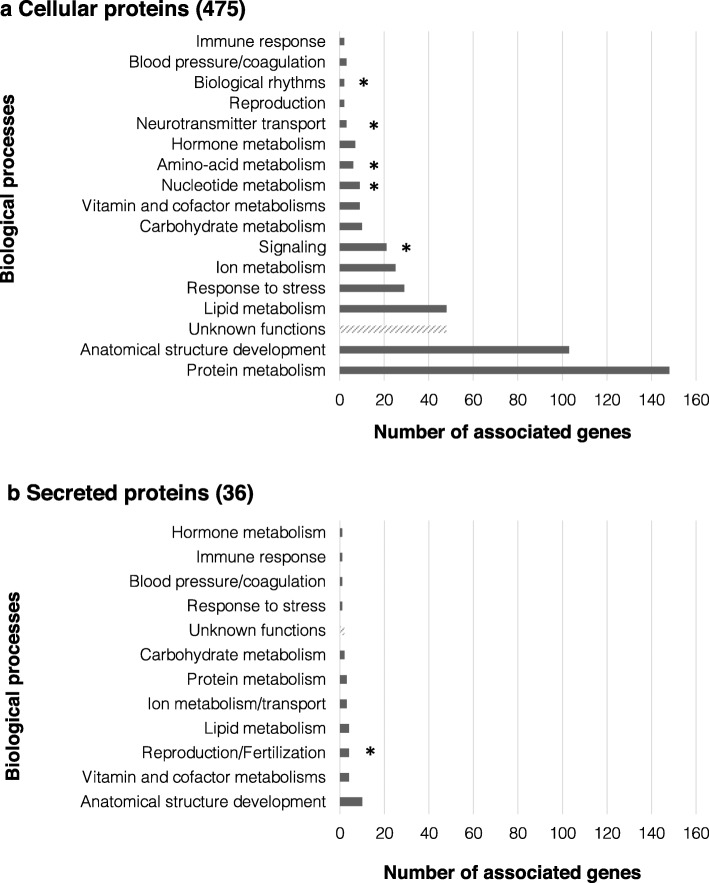
Functions associated with intracellular (**a**) and secreted (**b**) proteins. Proteins with unknown function are showed in hachured bars. Functions uniquely associated with one or the other group are signaled by an asterisk

Our data analysis revealed that 475 proteins are localized intracellularly, 36 are secreted from the liver and 5 proteins have an uncharacterized subcellular localization. The analyses of the putative functions of these proteins surprisingly highlight that some proteins within the cellular and secreted groups are complementary actors of common biological processes including reproduction, anatomical structure development, vitamin and cofactor metabolisms, carbohydrate metabolism, lipid metabolism, ion metabolism, protein metabolism, hormone metabolism, response to stress, blood pressure/coagulation, immune response (Fig. 2, Additional file 2, columns J-M). It is noteworthy that the non-secreted group contains additional biological processes that are indeed usually associated with intracellular processes: signaling, nucleotide and amino-acid metabolisms, but also biological rhythm and neurotransmitter transport (Fig. 2a, Additional file 2), while four secreted proteins are likely to have a specific role in fertilization (Fig. 2b, Additional file 2).

The major functions associated with cellular proteins encompass protein and lipid metabolisms, and anatomical structure development. Concerning secreted proteins, biological processes cover a number of various biological functions that might be associated with different physiological processes in other tissues/organs. It is also noticeable that 50 proteins have no assigned functions yet (48 cellular proteins and 2 secreted proteins, Fig. 2, Additional file 2).

#### Basic functions

Among the basic physiological processes associated with the liver of laying hens, we can find functions that are concomitant to an increased stimulation of the liver activity and egg yolk formation. Intracellular proteins are linked to hormone metabolism, reproduction, anatomical structure development (cell growth, cytoskeleton organization, cell shape, organ development), signaling, protein metabolism (transcription, translation, folding, transport, catabolism), lipid metabolism, and other increased metabolisms (nucleotide, amino-acid, ion, carbohydrate, vitamin and cofactor) (Fig. 2, Additional file 2, column J). Secreted proteins with high values of overexpression are known to be associated with egg yolk formation/fertilization (Additional file 2). Of the 36 overexpressed genes that are predicted to be secreted (Additional file 2, lines 491–526), only 18 are recovered in the yolk (Table 2) [13–15]. This observation suggests that 18 remaining secreted proteins may target other tissues than the ovary and the yolky follicles. As expected, vitellogenins, components of very low-density lipoproteins (apovitellenin, apolipoprotein B), and riboflavin-binding protein, which are highly abundant in egg yolk are also highly overexpressed in the liver of laying hens. It is noteworthy that many proteins that lack mammalian homologs (Table 1), are also abundant proteins of egg yolk, which supports their specific function in relation to the development of an embryo outside mother’s body, as opposed to mammals: riboflavin binding protein (RBP), vitellogenins (VTG1, VTG3), apovitellenin (APOV1), cathepsin E-A like (CTSEAL), avidin-related protein 6-like (LOC426220).

**Table 2:** Genes overexpressed in livers of mature hens, that are predicted to be secreted and whose protein products have been identified in egg yolk and/or vitelline membrane. EY, egg yolk; VM, vitelline membrane [13–15]. ML/IL ratio: ratio of gene expression in the liver of 38-week laying hens (ML) to the expression in the liver of 10-week juvenile pullets (IL)

Name [Gallus gallus]	Gene Symbol/ID	Localization	Ratio ML/IML	Subcellular location
riboflavin-binding protein	RBP/396449	EY;VM	66.92	Secreted
vitellogenin-3	VTG3/424534	EY;VM	45.88	Secreted
apovitellenin-1	APOV/396476	EY;VM	42.37	Secreted
cathepsin E-A-like	CSTEAL/417848	EY;VM	34.85	Secreted
vitellogenin-1	VTG1/424547	EY;VM	33.79	Secreted
WAP four-disulfide core domain 8	WFDC8/419301	EY	20.87	Secreted
zona pellucida sperm-binding protein 1	ZP1/395418	EY;VM	15.05	Secreted
avidin-related protein 6-like	LOC426220/426220	EY;VM	10.48	Secreted
lactadherin	MFGE8/415494	EY;VM	5.75	Secreted
transcobalamin-2	TCN2/429737	EY	5.47	Secreted
apolipoprotein B	APOB/396535	EY;VM	5.13	Secreted
ovochymase-1	OVCH1/769290	VM	4.01	Secreted
nidogen-1	NID1/395531	EY	2.77	Secreted
uncharacterized protein LOC421956	LINC00954/421956	VM	2.97	Secreted
fibulin-1	FBLN1/373979	EY	2.35	Secreted
thrombospondin-2	THBS2/414837	VM	1.53	Secreted
ceruloplasmin	CP/771940	EY	1.50	Secreted
vitamin D-binding protein	GC/395696	EY	1.48	Secreted

#### Newly identified functions

Besides these well-known functions, we proposed several additional functions.

First, the liver of laying hens appears as a peripheral clock tissue. Indeed, four genes related to nuclear clock genes have been identified in our study: period circadian clock 3 (PER3, fold change =1.73), nuclear receptor interacting protein (NRIP1, fold change = 1.62), nuclear receptor subfamily 1 group D member 2, (NR1D2, fold change =1.87, initially classified in the “lipid metabolism group”, Additional file 2) and activating transcription factor 4 (ATF4, fold change = 1.49, classified in the “protein metabolism” group, Additional file 2).

We also identified several genes related to hormone response/reproduction.

The prolactin receptor (PRLR) is highly overexpressed (fold change =6.84) as compared with juvenile hens, which suggests that the liver is likely to be strongly responsive to circulating prolactin.

Two proteins potentially stimulated by sex hormones have been identified as slightly overexpressed genes: the nuclear arginine and glutamate rich 1(ARGLU1, fold change 1.31) that is required for the expression/transcription of the estrogen receptor 1 target genes, and the progesterone receptor membrane component 2 (PGRMC2, fold change = 1.53), which is ubiquitous in mammals, integral to the membrane and that is known to be a receptor for steroids. We also noticed the high overexpression of prostaglandin F2-alpha receptor (PTGFR, fold change = 10.51), which initiates luteolysis following ovulation in mammals. Surprisingly, we identified several genes associated with the biosynthesis of steroid hormones. The cytochrome b5 reductase 2 (CYB5R2, fold change = 1.41) that is assumed to participate in steroid biosynthesis in human, being essentially testis-specific (Additional file 2, column G), is expressed in the liver of laying hens. Similarly, hydroxysteroid dehydrogenase like 1 (HSDL1, fold change = 1.32) has been shown to catalyze the metabolism of steroid hormones, thereby playing an important role in sex differentiation, the emergence and the maintenance of the secondary sexual characters. Finally, the sterol carrier protein 2 (SCP2, fold change = 1.25) may also participate in steroidogenesis as a sterol transporter. Altogether, these data question the partial contribution of the liver to steroid hormone biosynthesis in laying hens. In parallel, as a detoxifying organ, the liver is also likely to participate in steroids catabolism to ensure steady-state levels of plasma hormones. The presence of TEF (fold change = 1.46) and of a member of sulfotransferase family may contribute to such a process considering its high factor of overexpression (SULT, fold change = 5.47, Additional file 2, column D), although this gene currently lacks functional annotation in database (Additional file 2). Interestingly, this SULT homolog is found in birds and reptiles but not in mammalian species, which supports that SULT may have a specific role related to oviparous physiological specificities.

The functional annotation of secreted proteins in the circulating blood also revealed that some of them may play a role to assist oocyte fertilization. Three gamete interacting proteins containing zona pellucida domains were identified: zona pellucida sperm-binding protein 1 (ZP1, fold change =15.06), lactadherin (MFGE8, fold change = 5.75) and PREDICTED: uncharacterized protein LOC421956 isoform X5 (LINC00954, fold change = 2.97). Moreover, the ovochymase-1 (OVCH1, fold change = 4.01) is also assumed to favor sperm-egg interaction (Additional file 2). Remarkably, all these proteins are highly overexpressed in the liver of laying hens while they lack expression (or exhibit a very low expression) in the liver of human species (Additional file 2, column G).

Hormone regulators are also suspected to be associated to hen’s metabolism. The potential role of the liver to regulate thyroid hormone availability is evidenced by the overexpression of plasma glutamate carboxypeptidase precursor (CPQ, fold change = 8.37) that is secreted and that may play a role in the release of thyroxine hormones (T4/T3) from their thyroglobulin precursor. The overexpression of this protein by the liver of laying hens may be concomitant with the overexpression of membrane iodothyronine deiodinase 2 (DIO, fold change = 1.99), which triggers the deiodination of T4 (3,5,3′,5′-tetraiodothyronine) into T3 (3,5,3′-triiodothyronine) (Additional file 2). These two proteins are known to be essential for providing appropriate levels of T3 during critical periods of development. Both proteins are essentially expressed in the thyroid but not in liver in human (Additional file 2, column G).

The tremendous stimulation of liver activity and development observed at sexual maturity of hens, suggests compensatory mechanisms to counterbalance stress and its potentially deleterious over-activity. This hypothesis is corroborated by the numerous genes related to stress response and inflammation that have been identified in this study (30 genes, Fig. 2, Additional file 2, column J). Besides, we identified four overexpressed genes associated with blood homeostasis. These genes encode two membrane proteins (glutamyl aminopeptidase, ENPEP (fold change = 1.99), angiotensin II receptor associated protein, AGTRAP (fold change = 1.95) that participate in the renin-angiotensin system to regulate blood pressure. In addition, we identified the receptor activity modifying protein 2 (RAMP2, fold change = 1.69) that transports the calcitonin gene-related peptide type 1 receptor to the plasma membrane with which it acts as receptor for adrenomedullin, a potent hypotensive and vasodilator agent. The multimerin 1 (MMRN1, fold change = 1.87), which is secreted to play a role in the storage and the stabilization of factor V in platelets and in thrombin activation (coagulation) (Additional file 2) was shown to be upregulated in this study.

Interestingly, we identified several secreted proteins that could assist the formation of the specialized bone type known as medullary bone, whilst cortical bone production is minimal in laying hens. This physiological change is required at sexual maturity of laying hens as this medullary bone is a woven bone that provides a labile source of calcium that is essential for eggshell formation. We hypothesize that phosphoethanolamine/phosphocholine phosphatase (PHOSPHO1, fold change = 1.35) and bone morphogenic protein 7 (BMP7, fold change = 1.63) that both lack expression in the liver of human, and fibroblast growth factor 23 (FGF23, fold change = 1.78), which is almost exclusively expressed in the liver in human species, may be partly involved in bone remodeling.

#### Genes with no assigned functions

Among the 48 cellular proteins with no assigned functions, sulfotransferase (Gene ID 395933) has the highest fold change (fold change = 5.47). Concerning secreted proteins, only two proteins with still undefined functions are over-expressed: cathepsin E-A-like (CSTEAL, fold change = 34.85) and cysteine-rich with EGF-like domain protein 2 (CRELD2, fold change = 2.35). Five proteins have no defined subcellular localization (Additional file 2, column F).

## Discussion

**Fig. 3:**
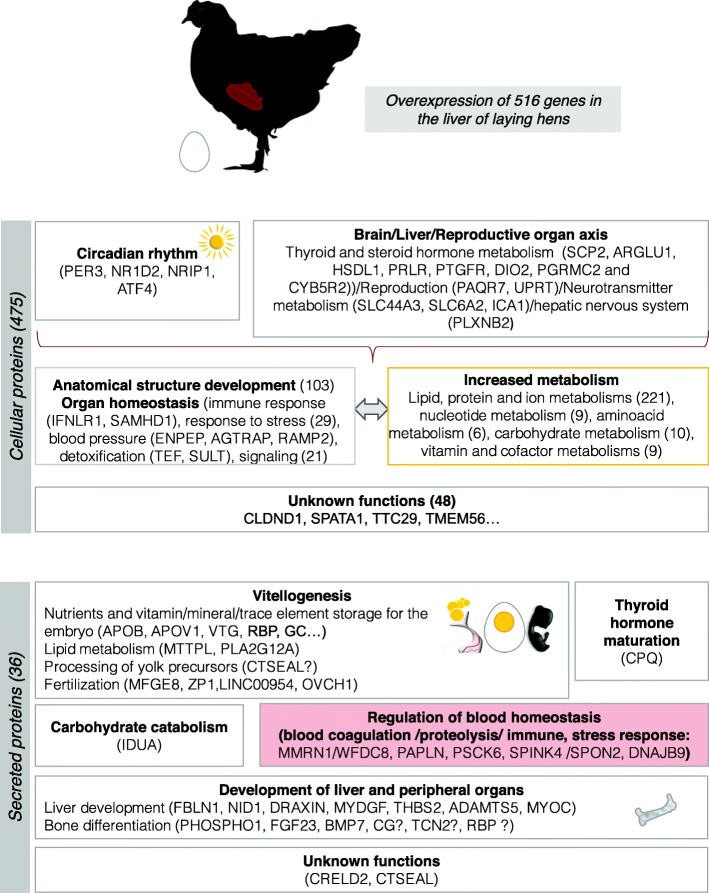
Drawing summarizing the physiological and metabolic impacts of sexual maturity in laying hens

The strong increase in circulating sex hormones at the onset of sexual maturity affects a variety of traits associated with reproductive functions (vitellogenesis), including secondary sexual characteristics [6] and organs’ metabolism. The aim of the present study was to describe the adaptation of the liver molecular repertoire at sexual maturity in the domestic fowl, by targeting over-expressed genes in sexually mature hens in comparison to juveniles, based on previous published data obtained by high-throughput approach [11]. This study endeavors to propose new aspects of the liver physiology at sexual maturity of chicken females, beyond the well-known lipogenesis and protein synthesis related to egg yolk formation (although these functions are also highlighted in the present work). The discussion starts with an overview of the interconnected biological processes assigned to proteins that are confined to the liver (cellular and membrane proteins) and that respond to various circulating metabolites, hormones, neurotransmitters, and ends with the functions of secreted proteins in yolk formation and likely other functions targeting peripheral organs/tissues. A schematic representation of the main conclusions of our functional analysis is proposed in Fig. 3.

### Sexual maturity in laying hens is associated with clock genes overexpression in the liver

Sexual maturity in bird females is triggered by light stimulation although the onset of sexual maturity also depends on the stage of the life cycle, environmental temperature, general health of pullets and adequate nutrition [16]. Thus, the circadian clock is a major regulator of a wide range of physiological functions including metabolism, sleep, body temperature, blood pressure, endocrine functions, and coordinates rhythmic gene expression in multiple tissues. In chickens, circadian/clock genes include PER, CRY, BMAL genes, which are expressed in several tissues including the thyroid gland, multiple oscillatory systems (the retina, the pineal and the hypothalamus) [17] but also reproductive tissues [18, 19]. The expression/activity of these genes/proteins is crucial to synchronize gene transcription/translation of key metabolic pathways thereby orchestrating the time course of physiological and behavioral processes. Preparation/training of these cellular clocks is achieved through exogenous daily inputs, including light (suprachiasmatic nucleus of the brain) and food (peripheral organs). Like many organs, the liver has an internal timing system, which adjusts physiological processes to rest/activity and feeding/fasting cycles throughout the day [20]. Such an effect of the diet on the expression of clock genes in the liver of laying hens and other peripheral organs (jejunum) have been published recently, showing that the expression of clock genes are trained in response to a specific sequence of feeding but also depends on the composition of the diet [21, 22]. Similar response to lighting and feeding programs on DNA synthesis and mitotic activity has been reported in the liver in the young chick [23, 24] but to our knowledge it has never been described in the liver laying hens. In the study published by Bourin et al. [11], to assess the impact of sexual maturity/physiology on liver transcriptome, 10-week pullets and 38-week laying hens were reared under the same environment with ad libitum access to water and food (same diet for pullets and laying hens) and with a cycle of 14 h of light and 10 h of dark. Samplings of liver for both pullets and laying hens were achieved within 1.30 to 3 h after light extinction (See Methods). The transcriptomic analysis of the liver of laying hens revealed an overexpression of PER3 gene (1.73 fold overexpression) but also four other clock-associated candidates that were not previously described for the chicken species: NRIP1, NR1D2, ATF4 (Fig. 3) and presumably MAPK9 (fold change = 1.57, additional file 2, column M). NRIP1 gene encodes the nuclear receptor interacting protein 1, that is localized in the nucleus, and that is a positive regulator of the circadian clock gene expression. It also modulates transcriptional activation by steroid hormones. It contains a Pro-X-Asp-Leu-Ser motif that is assumed to play an important role in circadian rhythmicity through regulation of protein interactions [25]. NR1D2 (Nuclear receptor subfamily 1 group D member 2, 1.87 fold overexpression) is a transcriptional repressor, which coordinates circadian rhythm and some metabolic pathways (lipid metabolism and inflammatory response) in several species. It essentially represses the expression of core clock components including ARNTL/BMAL1 and CLOCK genes. Similarly, studies on the activating transcription factor-4 (ATF4) have reported that this transcription factor is essential for the expression of clock genes including PER genes, via the modulation of cAMP dependent signaling [26]. Regarding MAPK9, it has been shown to phosphorylate clock ARNTL-BMAL1 heterodimer thus regulating circadian clock expression of specific genes [27]. It is also involved in the phosphorylation of a number of other transcription factors in response to physical stress or proinflammatory cytokines, to accompany cell proliferation, differentiation, migration, transformation and programmed cell death. It is noteworthy that five other clock genes spotted onto the microarray were not differentially expressed: CRY2 (*p* = 0.64), CLOCK (*p* = 0.928), NPAS2 (*p* = 0.087) while Bmal2 and Bmal1 were close to statistical significance (*p* = 0.07 and *p* = 0.015, respectively). The biological significance of such an overexpression in the liver of laying hens needs to be explored further and may depend on the reproductive status of animals (clock gene entrainment/physiological requirements associated with reproduction). Indeed, in mammals, pregnancy and the transition from pregnancy to lactation have been shown to regulate clock gene expression in the liver of mice [28, 29]. Because at sexual maturity the liver of birds is intimately associated with vitellogenesis, it might not be surprising to observe a regulation of clock genes expression in the liver of laying hens as compared with pullets.

### The liver of laying hens contributes to interorgan communication

In parallel to the expression of clock genes, the stimulation of photoreceptors will participate in the release of gonadotropin hormones (GnRH). In domesticated hens, GnRH was detected in the median eminence, with a downstream effect to enhance the release of gonadotropin releasing hormones LH and FSH by the anterior pituitary. These gonadotropin releasing hormones indirectly stimulate growth of the oviduct [30] following the increased secretion of estrogens [1]. In addition, in combination with testosterone, they regulate the ovarian growth, maturation of follicles and together with progesterone, the ovulation of mature follicles [31]. The liver was previously reported to express prolactin receptor (PRLR) [32], which is further confirmed in the study presented here (fold change = 6.84 in laying hens versus juvenile pullets). The substantial expression of prolactin receptor suggests that the liver constitutes a highly responsive organ to the circulating prolactin that increases considerably upon photostimulation [33, 34]. Such an overexpression of prolactin receptor of the liver to circulating prolactin may be concomitant to the effect of prolactin on follicular development and egg laying [35]. The binding of prolactin to its membrane receptor activates a number of signaling pathways resulting in the transcription of genes necessary for some tissue specific changes. Knowing that in other species, the depletion of prolactin receptor is associated with an accumulation of triglycerides in the liver [36] and that prolactin is also a regulator of gene expression [37], the high level of expression of prolactin receptor is quite coherent and is likely to accompany the increase in protein synthesis associated with lipogenesis at sexual maturity. However, the prolactin-inducible genes of the liver in laying hens remain to be identified to further appreciate the effect of circulating prolactin on the liver organ. Similarly, the over-expression of PTGFR (fold change = 10.51) may also suggest a major role of prostaglandin signaling at the hepatic level, in sexually mature hens. Prostaglandins have a preponderant role in the uterus, by controlling oviposition [5]. However, the biological significance of the high overexpression of this receptor in the liver remains to be elucidated. Additionally, we identified several genes associated with the biosynthesis/transport of steroid hormones (CYB5R2, HSDL1, SCP2), which questions the contribution of the liver to hormone biosynthesis (at a much lesser extent as compared with the ovary), and catabolism (SULT gene). A liver-gonad communication may be also mediated by PAQR7 (fold change = 6.33), PGRMC2 (fold change = 1.54) and UPRT (fold change = 1.84) localized in the plasma membrane and the nucleus. PAQR7 may form a complex with PGRMC2 (a responsive element to steroids), within the cytoplasm of the liver to regulate the antimitotic action of progesterone. This regulation is indeed necessary for liver growth. Such a PAQR7/PGRMC2 complexation and activity has been reported in human granulosa/luteal cells [38].

We also searched for a potential liver-brain-liver cycle. We found several genes that may help to further decipher how plasma compounds are sensed by the liver, and how this information is integrated by the liver and further processed via hepatic neuronal pathways to the brain or to other organs. The brain will in turn control the liver metabolism and that of other organs and tissues [39] via secretion of hormones and neurotransmitters in the blood. It has been established that the liver is innervated by an autonomous nervous system, and that the metabolic sensing function of the liver may also have repercussion on the brain activity and expression [39]. The expression/secretion of DRAXIN gene by the liver of laying hens (fold change = 3.09) that we observed, could play a role in liver innervation reorganization that is associated with its development. Indeed, the DRAXIN gene was found in brain and spinal cord, and acts on axon guidance in chicks [40]. In addition, the upregulation of PLXNB2 gene encoding a non-secreted protein in the liver could contribute to the regulation of neuronal migration to the liver at sexual maturity. A previous study pointed out that SVZ derived cells (generating neural stem cells) deficient for Plxbn2 exhibited a fast migration of neuroblasts compared to control conditions [41]. Moreover, interactions between brain and liver may rely on the upregulation of genes involved in neurotransmitter transport such as SLC6A2 (fold change =7.23), which is involved in norepinephrine and dopamine transport. SLC6A2 regulates many functions of the sympathetic system in human, learning, memory, mood, attention, stress and blood flow [42] (Fig. 3). Some other genes may participate in neurotransmitter transport. SLC44A3 (fold change = 2.37) that belongs to the choline-transporter like family and ICA1 (fold change = 3.66) are both described to be neurotransmitter transporters. All these data suggest some liver-brain-liver communications [39] in laying hens. Although no significant function is assigned to CRELD2 in Uniprot database (Fig. 3), the over-expression of this secreted protein may also strengthens the hypothesis of tight relations between the brain and liver, since this protein was reported to bind some subunits of the neuronal nicotinic acetylcholine receptors [43].

### The liver of laying hens undergoes increased protein synthesis

High amounts of proteins are synthesized by the liver to sustain the enhanced basal metabolism activity at the onset of sexual maturity of hens. The intense protein synthesis is attested by the high number of up regulated genes involved in protein metabolism (157 out of 516; 6 amino-acid metabolism/151 protein metabolism). The over-expressed genes are linked to many processes associated with protein synthesis: genes regulating DNA transcription (SOX7, fold change =4.56), ribosome biogenesis (RPF1, fold change =1.36), RNA splicing (RSRC1, fold change =1.36 and ZBTB8OS, fold change 1.35), mRNA translation (EIF2A, fold change =1.93). Nucleotide synthesis also increased in the liver at sexual maturity with GMPS (fold change = 1.32) and CMPK1 (fold change =1.54) genes regulating purine and pyrimidine synthesis, CMPK1 contributing to pyrimidine nucleotide transport as well as THUMPD2 (fold change = 1.43) to tRNA methylation. Genes involved in hepatic synthesis and transport of amino acids are up regulated at sexual maturity (ALDH18A1, fold change = 2.92 and SLC7A2, fold change = 1.48, respectively). Once synthesized, proteins are folded (chaperone proteins including HSPA5, fold change =2.98) and exhibit post-translational modifications as pointed out by the up-regulation of malectin (MLEC, fold change = 1.39) that regulates N-glycosylation of proteins for example [44]. The enhancement of genes regulating glycosyltranferases like GCNT4 (fold change =1.60), EXTL2 (fold change =1.41) and B3GNT2 (fold change =3.69) are also evidenced in the present study. These enzymes catalyze the transfer of one or multiple sugar residues to a wide range of acceptor molecules such as lipids, proteins, hormones, secondary metabolites, and oligosaccharides [45]. Other genes such as SEC16A (fold change =1.36) and SCAMP1 (fold change =1.78) contributing to the transport of secreted proteins are upregulated and might participate in the secretion of hepatic proteins in the bloodstream.

The increased metabolism of the liver at the onset of lay is paralleled with an increase of the liver weight [30] and can be driven by intracellular proteins, including OTC (fold change = 4.84), STMN1 (fold change = 2.42) and PTPN3 (fold change = 1.87). Members of the PTPs family (protein-tyrosine phosphatases) such as PTPN3 enzyme contribute to de-phosphorylation of proteins and are major regulators of fundamental cellular processes, including proliferation, differentiation, and cell-cell adhesion, especially in gastrulation during early embryonic development [46]. This enzyme could also contribute to cellular processes triggering liver development of laying hens. STMN1 over-expression in the liver of laying hens is conceivable, as stathmin plays a critical role in mitosis [47].

### The liver hyperstimulation requires the maintenance of homeostasis

As expected, many secreted proteins have been associated to anatomical structure development (FLBLN1, fold change = 2.34; NID1, fold change =2.77; DRAXIN, fold change = 3.08; MYFGF, fold change =1.34; THNS2, fold change =1.53; ADAMTS5, fold change =1.72 and MYOC, fold change =1.48) and are likely to contribute to the development and increased activity of the liver of laying hens.

Considering the high amount of hormones, lipids and proteins secreted in the blood stream at sexual maturity, it is not surprising that genes associated with the renin–angiotensin system are also overexpressed as regulators of blood pressure and fluid balance (ENPEP, fold change =1.99, AGTRAP, fold change = 1.94). ENPEP converts angiotensin II to Angiotensin III, which activates the angiotensin II receptor together with AGTRAP, thereby promoting vasodilation and protecting against hypertension [48, 49]. Similarly, RAMP2 (fold change = 1.69) that complex with calcitonin receptor-like receptor is important to regulate the activity of adrenomedullin, a hypotensive compound [50, 51]. AGTRAP overexpression has also been associated with insulin resistance [52]. This is particularly intriguing knowing that chickens are less sensitive to high plasma glucose concentration as compared with mammals, while maintaining similar insulin plasma levels [53]. MMRN1 may also contribute to regulate blood pressure as it is released during platelet activation to limit platelet dependent thrombin generation and thus clotting, in vivo [54]. The activity of these genes may all concur to limit deleterious effects associated with high blood pressure (Fig. 3). Similarly, the increased metabolic activity of the liver at sexual maturity also constitutes a risk of high accumulation of reactive oxygen species (ROS). ROS are produced in majority in the electron transport chain of mitochondria, but also in the cytoplasm by various enzymes. The anti-oxidant capacity of the liver constitutes an important factor that influences its function. A balance between ROS generation and the anti-oxidant system is essential to scavenge ROS and thus attenuate oxidative stress damage. In the present study, 6 members of the anti-oxidant system are over-expressed at sexual maturity (CAT, CYB5A, MSRB3, GLRX, STK25 and TXNDC11, Additional file 2) and could represent a signature of the increased metabolic rate of the liver. Moreover, although there is currently a lack of information related to CRELD2 gene in Uniprot database, many publications report that this gene is overexpressed upon stress and may constitute a novel mediator in regulating the onset and progression of ER-stress associated dysfunctions (which may occur during hyperstimulation of liver in laying hens) [55, 56]. Some secreted proteins may also promote cardiac myocyte survival and adaptive angiogenesis for cardiac protection. Such activity has been reported for secreted protein MYDGF (fold change =1.35) that is overexpressed by the liver at sexual maturity of hens. Some have shown that endothelial cells proliferation and capillary density were higher after reperfusion in wild type mice compared to Mydgf KO mice, highlighting the role of this protein in cardiac myocyte survival and angiogenesis [57]. The presence of such a protein in the bloodstream may contribute to protect heart from increased liver metabolism and subsequent protein and lipid secretion in the blood. In addition, we found several enzymes involved in the catabolism of steroids, proteins and hormones but also detoxifying molecules such as TEF (fold change = 1.46) and SULT (fold change = 5.47) genes, which may be paralleled with the major role of the liver in clearing toxic compounds from the blood [20], to maintain body homeostasis. Interestingly, TEF and a SULT homolog genes have been shown to be rhythmically activated through clock genes expression in mammals [58, 59]. Finally, three immune response related genes have been shown to be overexpressed in the liver of laying hens and may be markers for a chronic inflammatory environment (IFNRL1, fold change = 2.02; SAMHD1, fold change = 1.88 and SPON2, fold change =1.80) [60, 61].

### The increased basal metabolism of the liver of laying hens supports egg yolk formation

The first role of the liver at sexual maturity of hens is to synthesize and secrete yolk precursors that will be incorporated into the developing yolky follicles by endocytosis. Yolk precursors are transported to the follicular wall via the blood stream and are released near the basal lamina at the ovary site [62]. In birds, the dietary source of energy has a major influence on the metabolic pathway used for the lipid egg yolk synthesis. The main source of dietary energy in laying hen diets is based on carbohydrates. To support the hepatic de novo synthesis contributing to the daily production of 6 g of triacylglycerols that are exported to the egg yolk, a high uptake of glucose is required [63]. Different transcripts of glucose transporters of the GLUT family were identified in the liver of broiler chickens [64, 65]. In chickens, similar pathways could be effective in laying hen’ hepatocytes at sexual maturity to sustain liver glucose uptake for de novo lipogenesis. Although the GLUT4 gene is absent from the chicken genome, a homolog of GLUT4 named GLUT12 has been recently shown to be expressed in the liver of chickens [65]. INPP5K is over expressed in the liver of mature hens (fold change = 1.35) and is defined as a specific regulator of insulin signaling in skeletal muscle. This gene was found to negatively regulate insulin signaling and glucose uptake by inhibiting GLUT4 docking and/or fusion to the plasma membrane in a myoblast cell line [66].

The main egg yolk components secreted by the liver are Very Low Density Lipoproteins (VLDL) composed of triglycerides and cholesterol esters, which are surrounded by an envelope of cholesterol, phospholipids, apolipoprotein B and apovitellenin 1 [62]. These components are synthesized by the liver from acetyl-CoA, produced from pyruvate derived from dietary glucose, and incorporated into the citric acid cycle [67]. Acetyl-CoA is then converted into malonyl-CoA and its elongation contributes to the synthesis of fatty acids. In the present study (Additional file 2), many genes involved in fatty acid synthesis are over-expressed at sexual maturity (FAR1, ACSBG2, ELOVL2, HADHB, ELOVL1, PEX11A, ACSM3, LPPR5, ACPL2, PECR..., Additional file 2). Genes regulating the synthesis of the membrane components of the VLDL like phospholipids (PTDSS1, MTMR7, GPAM, PISD, ABHD6, ANGPTL3, MBOAT2, CHKA), cholesterol (HSDL1, NCEH1, SQLE) as well as lipoproteins apolipoprotein B (APOB) and apovitellenin 1 (APOV1) produced by the liver are all overexpressed in this study (Additional file 2). Similarly, the expression of some genes involved in the intracellular fatty acids binding and lipid transport [67] is also stimulated (FABP3, OSBPL3, TEX2, DBI, COL4A3BP, TTPA, MTTPL, Additional file 2). In parallel, genes underlying mechanisms of lipid degradation (PNPLA3, AIG1, ABDH2, PLA2G12A) as well as beta oxidation (HAO2, ACADL, DECR1, CPT2) are up regulated in the liver of sexually mature hens. Some other overexpressed genes regulate hepatocytes metabolism (OMA1, TMEM97, MBOAT2) and cholesterol homeostasis (TMEM97). Genes of the sphingolipid metabolism are also present in this study with KDSR, ARSD and ARSE as well as PCTP for the sphingolipids transport. These genes could be involved in the turnover of the hepatocyte cell membrane. This high amount of lipoproteins newly secreted in the blood have to be preserved from degradation until they reach their target tissue, namely the yolky follicles. This protein protection is likely to be achieved by ANGPTL3 and APOV1, a potent lipase inhibitor [68]. Altogether, these data highlight the multiple mechanisms involved in lipid metabolism [12]. In addition to VLDL, vitellogenins are also main yolk precursors. Their synthesis by the liver is mainly under the control of estrogens. VTG proteins constitute nutritional reserves that are stored in the egg yolk and, which are crucial for the development of non-mammalian oviparous vertebrates and invertebrates. VTG proteins contain phosvitin domains that chelate calcium, iron and other cations from the bloodstream. The binding capacities of VTG3 will provide trace elements and minerals for the developing embryo [62]. The synthesis and release of egg yolk proteins by the liver in the blood circulation, suggests the involvement of a protective system to prevent egg yolk proteolysis by circulating proteases, similarly to the activity of lipase inhibitor APOV1 that protects VLDL lipids. From our data, SPINK4 (fold change = 1.69) was over-expressed by the liver at sexual maturity. It encodes a serine peptidase inhibitor, Kazal type 4. No precise role of this protein was described so far, but the potent inhibitory activity of kazal-type protease inhibitors is well documented, suggesting that SPINK4 may protect egg yolk proteins from proteolytic degradation by blood proteases. Similar function can be hypothesized for PAPLN (fold change = 2.74) and PCSK6 (fold change = 2.20) but also WAP four-disulfide core domain protein 8 (WFDC8), a secreted protein that is highly overexpressed in the liver of laying hens (fold change =11.88). Concerning bird-specific CTSEAL (fold change = 34.85), this secreted protein share high protein sequence similarity with cathepsin D, which suggests that CTSEAL might assist cathepsin D in the processing of egg yolk precursors [69].

### The liver of laying hens sustains egg fertilization

Interestingly, four genes overexpressed in the liver at sexual maturity of hens (MFGE8, ZP1, LINC00954, OVCH2, Additional file 2) are prone to participate in oocyte fertilization. Fertilization includes sequential steps of species-specific sperm-egg binding, induction of the acrosomal reaction, sperm penetration through the oocyte, and the fusion of gametes. Fertilization in hens occurs in a funnel-like structure called the infundibulum that is localized in the upper part of the oviduct and that receives each sequentially ovulated follicle. ZP1, MFGE8 and LINC00954 all contain zona pellucida domains. ZP1 (fold change = 15.06) is synthesized by the liver and is transported to the ovary via the blood to be inserted in the perivitelline membrane surrounding the oocyte [70]. Little is known regarding the involvement of MFGE8 (fold change = 5.75) and LINC00954 (fold change = 2.97) in fertilization, but the fact that both proteins are highly expressed and secreted, and that they possess zona pellucida domains are consistent with such a hypothesis. Moreover, recent studies have revealed that MFGE8 may be one substrate of proteasomal proteolysis during fertilization [71]. OVCH1 (fold change = 4.01) is a specific protease of the vitelline membrane that was found to be a key factor mediating sperm-binding to the oocyte in Xenopus and mammals [72]. This protease is involved in the maturation and proteolysis of major glycoproteins composing the zona pellucida [72]. It role in avian species has not been investigated yet.

From our integrative analysis, it seems that the proteins secreted by the liver at sexual maturity of hens not solely target the yolky follicles but may also have some other physiological sites. As mentioned in the introduction, sexual maturity also affects secondary sexual characteristics such as the comb size that is a sexual ornament, and bone structure of the hen.

### The liver of laying hens may participate in the establishment of secondary sexual characteristics

Old studies have suggested a link between thyroid and comb development [73] considering that thyroid hormones are involved in the regulation of seasonal reproduction in birds [74] and other animal species [75]. Interestingly, we found several overexpressed genes at the sexual maturity of hens, including one that is highly expressed and secreted (CPQ, fold change =8.37). CPQ is a carboxypeptidase that plays an important role in the hydrolysis of circulating peptides and that is possibly involved in the release of thyroxine hormone from its thyroglobulin precursor [76]. Similarly, the gene encoding iodothyronine deiodinase 2 (DIO2) was found to be over-expressed in the liver of mature compared to juvenile hens (fold change = 1.99). This overexpression may result from differences in food intake when comparing laying hens to pullets as the expression of this enzyme was shown to be regulated depending on food availability [77] and likely during the dark phase when the metabolism of hens is minimal. This enzyme is involved in the conversion of the prohormone thyroxine (T4) to the biologically active thyroid hormone triiodothyronine (T3) [78]. This result highlights a possible additional production site of T3 behind the mediobasal hypothalamus [79]. It has also been proposed that T3 stimulates hepatic malic enzyme activity contributing to lipogenesis [80].

During sexual maturation, the increase of calcium retention and medullary bone formation are the main adaptations to support eggshell formation. Medullary bone formation is induced by estrogens and testosterone during sexual maturation of pullets, modifying the osteoblast function to produce the medullary bone. This latter bone constitutes a labile source of calcium that will be mobilized over the dark period of the ovulatory cycle [7] concomitantly to the increase of phosphorus in the plasma. It has been shown previously that the expression of FGF23, a peptide hormone produced by bone, is regulated by dietary/plasma phosphorus and that this regulation is different in the liver as opposed to the bone [81, 82]. Additional organs are also able to synthesize FGF23, including the liver of laying hens [83]. In the present study, we confirm that the liver of laying hens expresses a higher level of FGF23 (fold change = 1.78) that might assist the development of medullary bone by regulating circulating phosphorus [84–86]. The over-expression of secreted PHOSPHO1 (fold change = 1.35) could also be a signature of this bone remodeling during egg production. PHOSPHO1 was first studied in chicks where it is expressed at higher levels in mineralizing compared to non-mineralizing tissues [87]. In mice, this protein was proposed to participate in the generation of inorganic phosphorus that is required for the hydroxyapatite crystals formation during the mineralization phase of the bone. Deletion of PHOSPHO1 in mice conducted to a hypomineralization status [88]. Besides, BMP7 (fold change = 1.63) may also participate in bone remodeling [89], although this protein also plays a pivotal role in energy metabolism [90] and liver regeneration [91].

### The liver may supply vitamin-binding proteins to convey essential vitamins to peripheral organs including the yolky follicles

Many vitamin binding proteins are stimulated at sexual maturity to favor the storage of vitamins in egg yolk. The vitamin D binding protein [92], the retinol and riboflavin binding proteins [93] are stimulated by estrogens at sexual maturity, which is consistent with the overexpression of vitamin D, riboflavin but also cobalamin binding proteins genes in mature hens (Table 2). These vitamin-binding proteins might have additional tissue targets considering that riboflavin, vitamin D [94], and cobalamin can also play a role in bone development and remodeling. Deficiencies in those vitamins induce leg problems including paralysis [95] and cobalamin deficiency may lead to decreased bone mass following increased osteoclast formation [96, 97]. Knowing that vitamins are incorporated in the diet, we hypothesized that some of the highly overexpressed vitamin-binding proteins in the liver at sexual maturity (RBP, TCN2, GC that transport riboflavin, cobalamin and vitamin D, respectively) may also facilitate transport/storage of these essential vitamins to accompany bone remodeling. In parallel, the increase of conversion of T4 to T3 at sexual maturity suggested by the over-expression of DIO2 and CPQ may also contribute to such physiological changes as T3 was shown to regulate bone skeletal development and remodeling [98, 99]. However, such hypotheses require further investigation.

### Many overexpressed proteins of the liver of laying hens lack functional information

Last but not least, it has to be underlined that several genes that were shown to be overexpressed in this study have no mammalian homologs (Table 1). It is now assumed that identifying lost genes in some species has great insight to uncover the molecular mechanisms underlying the evolution of a wide range of adaptive phenotypes. Gene loss is a mechanism that has likely contributed to adaptive evolution in several mammals and it is now well established that it may be the consequence or the cause of phenotypic evolution [100]. As examples, the loss of vitellogenin genes in mammals is paralleled with evolution to lactation [101]. A better characterization of these genes may help to understand some phenotypic specificities of the avian model. Moreover, in contrast to mammals, the female chicken is heterogametic (ZW) and the male is homogametic (ZZ). We still do not know the biological significance of the presence of SPIK4 protease inhibitor and LOC426220 avidin-like protein (biotin binding) in sexual chromosomes Z and W, respectively. Regarding LOC426220, all other avidin-like molecules are localized on chromosome Z (AVD, 396260; AVDL, 431660, avidin-like NC_006127.5 (9,136,528..9137510), AVR2 ID: 395367) [102]. These data suggest some regulation that are sex and species-specific.

Although the deep integration of under-expressed genes may also help to better appreciate the mechanisms supporting the overexpressed genes and the new reproductive functions, the present work focusing on over-expressed genes in the liver of laying hens reveals new research avenues to study the physiological changes associated with sexual maturity of laying hens.

## Conclusions

This integrative study highlights the importance to re-analyze/update data with regards to new genome assemblies, experimental data published in literature on chickens and hens, and having in mind physiological changes associated with sexual maturity. The present analysis confirmed known functions (lipid metabolism, lipogenesis, protein synthesis, organ homeostasis), while specifying some molecular actors and mechanisms. Interestingly, it also revealed new functions and the identification of proteins participating in circadian rhythm, in the brain/liver/reproductive organs axis and proposed some potential role in fertilization and the development of peripheral organs (bone remodeling).

## Methods

Data analyses and integration used to write the article are available as additional files 1 and 2. In the experiment published by [11], the diet and the light/dark cycle as well as the rearing environment were the same for pullets and laying hens. Animals had free access to water and food. Samplings of liver were performed between 2:30 PM and 4:00 PM, about 1 h30 to 3 h after light extinction for both pullets and laying hens.

### Data update

The list of overexpressed genes in the liver of laying hens were retrieved from NCBI Gene Expression Omnibus (GEO; http://www.ncbi.nlm.nih.gov/geo/) under accession number GSE35595 [11]. The accuracy of gene ID and names and uniprot accession numbers were checked using NCBI database (https://www.ncbi.nlm.nih.gov/gene/) and Ensembl (http://www.ensembl.org/Gallus_gallus/Info/Index) (Additional file 1; GRCg6a (GCA_000002315.5) assembly).

### Comparative analyses of gene expression with human tissues

To perform comparative analyses with human species and to better appreciate tissue specificity of each candidate genes, we used the information available in each NCBI gene file using corresponding gene symbol and the keyword “human”. The generated gene file contains information about tissue expression based on RNA-sequencing performed on 95 human individuals representing 27 different tissues [103] (Additional file 2; column G).

### Analyses of post translational modifications

Subcellular localization of candidates genes were retrieved from Uniprot website (http://www.uniprot.org). These genes were further classified as secreted or cell proteins (intracellular proteins/integral components of membranes including plasma membrane). TMHMM Server, v. 2.0 (http://www.cbs.dtu.dk/services/TMHMM/) was used to predict transmembrane proteins and the number of transmembrane domains composing protein sequences (Additional file 2; column H).

### Functional annotation of genes

Functional annotation of genes was performed manually based on the information available in Uniprot files (Additional file 2, column M). Suggested functions for each candidate protein were summarized in Additional file 2, column J. This information combined to our known expertise on the physiology of laying hens were used to define 17 functional terms (Additional file 2, column J): biological rhythms; hormone metabolism; reproduction; anatomical structure development; signaling; nucleotide metabolism; amino-acid metabolism; lipid metabolism; protein metabolism; vitamin and cofactor metabolisms; carbohydrate metabolism; ion metabolism; response to stress; blood pressure/coagulation; immune response; neurotransmitter transport; unknown functions.

## Supplementary information

**Additional file 1.** Gene annotation of published data [11] based on current chicken genome databases (GRCg6a (GCA_000002315.5) assembly, submitted by the Genome Reference Consortium on April 2018).

**Additional file 2.** Integrative analysis of overexpressed gene in the liver of laying hens.

## Data Availability

The microarray data was previously deposited in the NCBI Gene Expression Omnibus (GEO) data repository under series accession number GSE35595 (https://www.ncbi.nlm.nih.gov/geo/query/acc.cgi?acc=GSE35595), as described previously [11].
